# Endoplasmic Reticulum Stress, Genome Damage, and Cancer

**DOI:** 10.3389/fonc.2015.00011

**Published:** 2015-02-03

**Authors:** Naomi Dicks, Karina Gutierrez, Marek Michalak, Vilceu Bordignon, Luis B. Agellon

**Affiliations:** ^1^Department of Animal Science, McGill University, Montréal, QC, Canada; ^2^Department of Biochemistry, University of Alberta, Edmonton, AB, Canada; ^3^School of Dietetics and Human Nutrition, McGill University, Montréal, QC, Canada

**Keywords:** carcinogenesis, cell death, chromatin damage, coping responses, DNA breaks, endoplasmic reticulum, unfolded proteins

## Abstract

Endoplasmic reticulum (ER) stress has been linked to many diseases, including cancer. A large body of work has focused on the activation of the ER stress response in cancer cells to facilitate their survival and tumor growth; however, there are some studies suggesting that the ER stress response can also mitigate cancer progression. Despite these contradictions, it is clear that the ER stress response is closely associated with cancer biology. The ER stress response classically encompasses activation of three separate pathways, which are collectively categorized the unfolded protein response (UPR). The UPR has been extensively studied in various cancers and appears to confer a selective advantage to tumor cells to facilitate their enhanced growth and resistance to anti-cancer agents. It has also been shown that ER stress induces chromatin changes, which can also facilitate cell survival. Chromatin remodeling has been linked with many cancers through repression of tumor suppressor and apoptosis genes. Interplay between the classic UPR and genome damage repair mechanisms may have important implications in the transformation process of normal cells into cancer cells.

## Introduction

**Figure 1:**
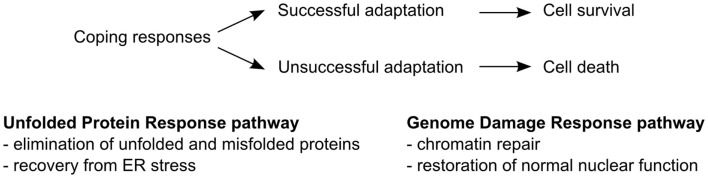
**Coping response mechanisms**. The unfolded protein response (UPR) pathway operates to restore correct folding of proteins and recovery of the ER from stress. The genome damage response (GDR) pathway enables repair of damaged DNA, histones, and other DNA binding proteins and restores normal nuclear function.

Cells in the body are continuously exposed to a dynamic environment dictated by the metabolic and nutritional status of the organism. Certain instances, such as exposure of the organism to nutrient excess or deprivation, extremes in temperatures, xenobiotics, and radiation, cause damage to cellular components and disruption of cellular processes. It has long been recognized that cells are adept at compensating for changes in their environment by altering certain cellular processes. The mobilization of such coping mechanisms is designed to maintain or recover proper function, overcome stressful conditions, and increase the chance for survival (Figure 1).

In the past several years, there has been increasing evidence linking endoplasmic reticulum (ER) stress with development of diseases, including certain types of cancers (1–5). In the case of cancers, especially non-inherited cancers that arise from genome damage, the cells capitalize on the ER stress response, which may be adaptive and advantageous at the cellular level, but deleterious to the organism. In this review, we discuss ER stress and genome damage in relation to cancer development. We provide observations supporting a link between different corrective strategies that cells adopt, which may lead to malignancies.

The accumulation of unfolded and misfolded proteins disrupts ER homeostasis and leads to the activation of the classic coping mechanism termed the unfolded protein response (UPR) (Figure 1). The UPR is initiated by the molecular chaperone glucose-regulated protein 78 (GRP78). GRP78 not only binds to the misfolded and unfolded proteins, but also regulates the transmembrane ER stress sensors, namely protein kinase RNA like ER kinase (PERK), inositol-requiring protein 1α (IRE1α), and activating transcription factor 6 (ATF6) (6–8).

Each ER stress sensor activates a separate arm of the UPR to facilitate immediate changes to a set of cellular functions designed to temporarily arrest general protein synthesis, and to produce active transcription factors that ultimately facilitate correct protein folding, degradation of proteins that cannot be properly processed, and regain of ER function. Under extreme conditions, these strategies may not be sufficient to alleviate the ER stress and thus require the removal of the malfunctioning cells. In such cases, cells undergo controlled cell death by activation of the apoptotic pathway. In some situations, certain adaptive strategies provide these cells with a selective growth advantage over other cells (Figure 1). This selective advantage could permit cells to survive and propagate even under chronic ER stress.

## ER Stress and Cancer

The high proliferative rates and inadequate vascularization of solid tumors culminate in a very unfavorable microenvironment. The low pH, low oxygen tension, and low nutrient supply result in an accumulation of misfolded proteins and ER stress, which could signal cell death (9, 10). Cancer cells, however, have developed a capacity to survive these extreme conditions, despite the presence of ER stress, through modulation of the UPR response (11–14).

It has been observed that GRP78, a dominant regulator of the ER stress response, is increased in a variety of cancer types including breast, brain, lung, colon, prostate, skin, and some other malignancies (2, 12, 15–20). This chaperone is associated with prolonged cell survival, mainly by preventing ER stress-induced apoptosis and thereby promoting cell malignancy, metastatic development, and resistance to anti-cancer agents (12, 14, 21, 22). High levels of GRP78 are also associated with rapid proliferation and malignancy of tumors (12, 14). In breast cancer cells that express estrogen receptor α [NR3A1], the estrogen-mediated increase in GRP78 abundance confers improved resistance to ER stress and cell proliferation, both of which can be decreased through siRNA-mediated knockdown of estrogen receptor α (12). Similarly, up-regulation of GRP78 has been shown to increase growth of a glioma cell line whereas its down-regulation inhibits tumor development (14). The reduction of GRP78 in glioblastoma cell lines and solid tumors treated with a chemotherapeutic agent increased the expression of CHOP and caspase 7, leading to cell apoptosis and inhibition of tumor formation (11, 14). Moreover, the antitumor agent HKH40A decreases GRP78 not only at the transcriptional level but also at the protein level by directly binding GRP78 to facilitate its degradation (11). Based on these characteristics, GRP78 is considered as a biomarker of cancer progression (21).

The components of the UPR pathway have also been implicated in cancer (2, 13, 21, 23). Mutations in IRE1α have been found in some human malignancies (24, 25). Under hypoxia, the effector of the IRE1α pathway, spliced XBP1 (XBP1s), is one of the factors involved in tumor growth and survival. It promotes cancer cell survival under low oxygen conditions by forming a transcriptional complex around hypoxia-inducible factor-1, a major gene regulator under hypoxic conditions (26). This transcription factor is also involved in human breast tumorigenesis as well as in the progression of triple negative breast cancer (26, 27). Similarly, the PERK pathway can contribute to cell survival and growth through ATF4, a transcription factor that induces pro-survival genes (28, 29). ATF4 is overexpressed in solid tumors and is essential for tumor cell survival in various mouse and human cancers whereas elimination of ATF4 in cancer cells induces apoptosis (29). PERK can also facilitate tumor growth by upregulating vascular endothelial growth factor (VEGF) and thereby inducing angiogenesis in tumors (28). Tumors derived from PERK-deficient mouse embryonic fibroblasts are considerably smaller compared to those derived from wildtype embryonic fibroblasts as a result of their impaired ability to stimulate angiogenesis (28).

Despite ample examples suggesting that the activation of the UPR is essential to cancer cell survival and tumor development, there are also indications that ER stress may provide protection against cancer (3, 30, 31). In particular, it has been shown that XBP1 is protective against intestinal tumorigenesis (3). Prostatic cancer cells have been shown to produce high levels of UDP-*N*-acetylglucosamine pyrophosphorylase 1, which reduces ER stress in these cells and facilitates their growth (30). The flavonoid baicalein has also been shown to induce ER stress in hepatocellular cancer cells, resulting in increased apoptosis (31). Interestingly, in this same study, increased IREα and eIF2α activation provided a survival advantage to theses cancerous cells. This finding highlights the paradoxical role of the UPR in cancer and our incomplete understanding of how signaling pathways may favor cell death or survival under different conditions (32–34). Whichever the outcome produced by ER stress, it is clearly apparent that the UPR plays a critical role in cancer biology.

## Genome Damage and Cancer

Genome damage can be caused by a number of endogenous and exogenous genotoxic factors, including reactive oxygen species, altered cell metabolism, xenobiotics, and radiation (35, 36). These factors lead to DNA strand breaks, collapsed DNA replication forks, and damage to histones as well as other DNA-binding proteins (35). In response to chromatin damage, cells can establish a genome damage response (GDR) to repair damage to both DNA and nuclear proteins, adapt to genome damage, and reestablish nuclear function (Figure 1). Adaptation to genome damage can lead to cell survival but also chromatin alterations, which may have severe consequences for tissue function and physiology (36).

**Figure 2:**
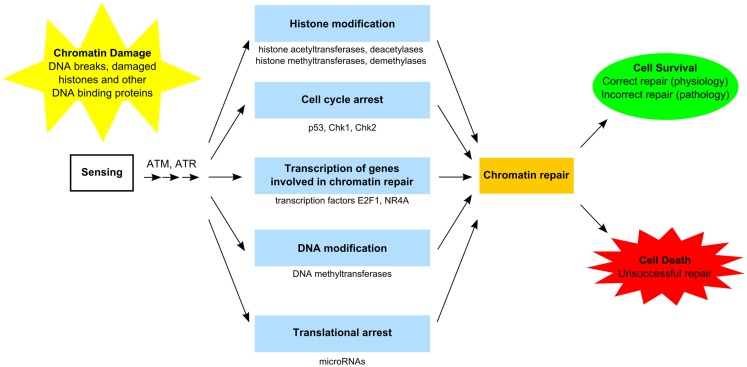
**Functional components of the GDR pathway**. Sensing of damaged DNA, histones, and other DNA-binding proteins results in activation of specific kinases [e.g., ataxia telangiectasia mutated (ATM) and ataxia telangiectasia and Rad3-related (ATR) proteins] (37–40). Access to damaged chromatin is facilitated by histone modification involving histone acetyltransferases/deacetylases and histone methyltransferase/demethylases as well as by DNA modification involving DNA methyltransferase (37, 38, 48–50). Certain transcription factors (e.g., E2F1, NR4A) stimulate genes involved in chromatin repair (45). Translational arrest is facilitated by microRNAs (e.g., mIR-155, miR-18a) (51, 52). Cell cycle arrest (e.g., via p53, Chk1, Chk2) may be required in order to complete chromatin repair (41–44). Unsuccessful chromatin repair due to extensive damage commits the cell to die. Successful chromatin repair enables cells to survive and restore normal function. In certain cases, incorrect repair escapes quality control surveillance and leads to altered cell function, which may provide the cell with a survival advantage, but manifest as pathology at the organismal level.

The GDR is orchestrated by several factors encompassing sensors, transducers, and effectors proteins (Figure 2), which require post-translational modification and accumulation of proteins to assemble multiprotein foci at the sites of DNA lesions (37–39). In general, activation of GDR involves temporary cell cycle arrest, local inhibition of transcription, and relaxation of chromatin to facilitate repairs. This process requires post-translational modification of proteins including the activation of the kinases ataxia telangiectasia mutated (ATM) and ataxia telangiectasia and Rad3 related (ATR), which phosphorylate transducer proteins at the damages sites, including the histone H2A.x (H2AX139ph), which anchors some important effector proteins required for damage repair and cell cycle arrest (37–40). ATM and ATR also activate the serine–threonine checkpoint effector kinases, Chk1 and Chk2, which regulate a number of proteins involved in transcription, cell cycle progression and apoptosis, including the tumor suppressor protein p53 and BRCA1 (41, 42), and the cell cycle regulator proteins Cdc25 and Wee1 (43, 44). Phosphorylation of transcription factors, notably E2F1, NR4A, ATF2, and Sp1, also facilitate DNA repair in a transcription-independent fashion, by direct interaction with damaged DNA, and subsequently the co-localization of other DNA repair proteins (45). In addition to post-translational modifications, genotoxic lesions and DNA damaging agents can also trigger nucleosomal remodeling via eviction of resident histones and reincorporation of new histones into the reassembled nucleosomes after damage repair (37, 46–48).

Local transcriptional arrest associated with GDR may be transient or stable (49, 53). Linked to this arrest are numerous histone modifications, including hypoacetylation of histone H4, increased histone H3K9me3 and H3K27me3, and decreased histone H3K4me3 (49, 50). Histone chaperones, which transfer histones to the nucleosomes, are required for transcriptional reinitiation following DNA damage (49, 54). GDR also involves the participation of ATP-dependent chromatin remodeling complexes, including the switch/sucrose non-fermentable (SWI2/SNF2), imitation switch (ISWI), inositol requiring 80 (INO80), and chromodomain helicase DNA-binding protein, which mediate nucleosome sliding and histone displacement promoting access for DNA repair proteins (37, 38, 48).

Non-coding RNAs are also known to affect DNA repair and genome instability. Indeed, DNA damage responsive microRNAs have been shown to be misexpressed in cancer cells and to affect chemotherapy sensitivity (55–57). It has also been shown that down-regulation of Dicer and Ago2, two essential microRNA processing components, reduced cell survival and checkpoint response after UV-induced DNA damage (58). Moreover, the miR-18a was shown to downregulate ATM expression, reduce DNA damage repair, and sensitize breast cancer cells to γ-irradiation treatment (51). Also, up-regulation of the miR-24 decreases H2AX and renders cells more vulnerable to DNA damage induced by γ-irradiation and genotoxic drugs (59). Another microRNA, miR-155, was shown to reduce the levels of RAD51, a recombinase required to repair double strand breaks by DNA homologous recombination, and consequently decreased DNA repair and enhanced sensitivity to ionizing radiation in human breast cancer cells (52).

## UPR and GDR Crosstalk

There is accumulating evidence suggesting that ER stress and GDRs are intertwined. Indeed, ER stress induced with tunicamycin treatment or glucose deprivation decreases genomic DNA damage repair by stimulating proteasomal degradation of Rad51 (60). On the other hand, down-regulation of PERK enhances DNA damage repair in irradiated cancer cells (61). Interestingly, induction of ER stress recruits the histone acetyltransferase p300 to the GRP78 promoter and this correlates with increased histone H4 acetylation and GRP78 gene expression (62). Increased GRP78 transcription is associated with the recruitment of arginine histone methyltransferase, PRMT1 (62). It was suggested that arginine methylation of MRE11 by PRMT1 regulates the activity of MRN complex, which is required for proper DNA damage checkpoint control (63). Therefore, it appears that increased GRP78 transcription from ER stress can also facilitate DNA damage repair. These contradictory effects further demonstrate our incomplete understanding of the stress signaling pathways and how they interact to determine cell fate. However, it also illustrates how ER stress can cause chromatin remodeling and affect the GDR pathway. If GDRs are impaired by alterations in the UPR, this can affect DNA integrity and subsequently increase risks of carcinogenesis.

Signaling from both ER stress and DNA damage also appear to result in similar chromatin remodeling changes to respond to cellular insults. Increased H3K14 acetylation as a consequence of ER stress has been observed, and this can activate the expression of other target ER stress response genes (64). Similarly, GDR results in increased H3K14ac, which promotes the binding of BRG1, an ATPase component of SWI2/SNF2 complex, to H2AXph139 at the sites of DNA damage enabling chromatin remodeling for DNA repair (65, 66). Phosphorylation of the histone H2Ax also enables recruitment of other chromatin remodeling complexes including INO80 and SWR1, and the histone acetyltransferase complex NuA4 to facilitate DNA repair (67–70). Therefore, H3K14ac and H2AXph139 seem to be important in connecting ER stress and GDR.

Chromatin remodeling has also been shown to occur as a result of hypoxia and heat stress, two common causes of ER stress that also have effects on GDR (71). Hypoxia-induced ER stress leads to global deacetylation and methylation of histones in the proximity of genes involved in the hypoxia-inducible factor-1-mediated response (72–74). This facilitates transcription of the genes needed for an adaptive response to hypoxia (75, 76). Yet, there is evidence confirming that hypoxia can lead to defective DNA repair, genomic instability, and consequently, to cellular transformation (76). In addition, it has been well documented that chromatin remodeling in response to heat stress results in increased transcription of heat shock proteins (77). These proteins have been shown to reduce accumulation of H2AXph139, decrease DNA damage repair, and increase radiation sensitivity and genome instability (78, 79).

While there is evidence demonstrating crosstalk between the UPR and GDR, it is not well understood at this time. Increased reactive oxygen species appears to be a common by-product of most cellular insults, ER stress, and DNA damage included (80). Oxidative stress can modulate multiple signaling pathways through activation of common transducers and transcription factors (81).

## Role of UPR and GDR in Carcinogenesis

Classically, the development of cancer is largely associated with inherited or acquired mutations of specific genes that regulate cell cycle, proliferation, and apoptosis (82, 83). However, similar effects can be seen with epigenetic changes, which, alone or associated with genetic mutations, can alter the expression of tumor suppressor genes (84–87). There are many examples of chromatin changes that lead to cancers. Hypermethylation of the DNA repair gene BRCA1 has been associated with both breast and ovarian cancer (88–90). Aberrant promoter methylation of the Kelch-like-ECH-associated protein 1 gene, which codes for an adaptor protein involved in degradation of cell survival and anti-apoptosis gene products, has been linked to a poorer prognosis and increased carcinogenesis in breast cancer patients (91). Hypermethylation of tumor suppressor genes has been observed in renal carcinomas and hematopoietic cancers (92–94). Hypermethylation of the cell cycle regulation gene RB1 and cyclin-dependent kinase inhibitor genes, CDKN2B and CDKN2A, which are, respectively, associated with the ocular tumor, retinoblastoma (95), and various leukemias and lymphomas (93). Histone deacetylation has been associated with a more aggressive form of acute myeloid leukemia (AML) through its repressive effect on the tumor suppressor gene death-associated protein kinase 1 (96). AML has also been associated with changes in histone methylation patterns (97). Finally, chromatin remodeling agents, including inhibitors of histone deacetylases, histone lysine demethylases, and DNA methyltransferases, have been tested for the treatment of various cancers (94, 98–102).

Since both ER stress and GDR coping mechanisms affect chromatin remodeling and DNA repair, adaptations based on these mechanisms could lead to emergence of malignant cells with self-renewal properties due to both genomic and epigenomic alterations. For example, hypermethylation of promoter regions around ER stress response genes have been implicated in the development of alcohol-induced liver cancer (103). GRP78-deficient mice fed large quantities of alcohol throughout their lives show high incidence of hepatic tumors, and correlate with hypermethylation of ATF6, which upregulates genes involved in ER-associated degradation to deal with the accumulation of misfolded proteins (103). Also, increased GRP78 stimulates the VEGF receptor 2 and subsequently VEGF-induced endothelial cell proliferation, which facilitates angiogenesis and tumor survival and growth (104–106). The apparent contradictory effect on neoplasticity as both inhibition and promotion of cancer progression, predicted by GRP78 abundance, suggests that the nature and context of coping response activation are important determinants of the outcome.

Acetylation of H3K14 has also been implicated in cell survival and carcinogenesis, both with respect to the UPR and GDR. Increase in H3K14 acetylation in response to ER stress results in stimulation of transcription, promoting cell survival (64). Increased H3K14ac during GDR enhances access of BRG1 to the sites of DNA damage to promote chromatin remodeling required for DNA repair (65, 66). However, in addition to promoting DNA repair, BRG1 has been associated with cancer development. For example, BRG1 was shown to impair the recruitment of BRCA1 to DNA damage sites, which is important in DNA damage repair and in the maintenance of genomic stability (107); to activate the melanoma inhibitor of apoptosis gene (108); and to support oncogenic transcriptional program, including Myc (109), for the survival of leukemic cells (110). Finally, chromatin changes in response to genotoxic conditions have been shown to alter the regulation of the Hedgehog–Gli signaling pathway, which has been implicated in genome instability and in several types of cancers (111–113).

## Summary

Coping mechanisms are designed to correct, minimize, or overcome damage caused by harsh environments, and promote cell survival. The UPR pathway is mobilized in response to the accumulation of unfolded proteins and to ultimately regain ER homeostasis. Similarly, the GDR pathway operates in response to chromatin damage and to restore normal nuclear function. Some adaptive strategies allow cells to overcome defects in cellular function through metabolic adaptation and gain a survival advantage, such as in certain types of malignancies. A better understanding of the interplay between UPR and GDR pathways may provide new insights into the pathogenesis of cancers, which could give rise to more effective anti-cancer therapies.

## Conflict of Interest Statement

The authors declare that the research was conducted in the absence of any commercial or financial relationships that could be construed as a potential conflict of interest.
